# An urgent need for improving thalassemia care due to the wide gap in current real-life practice and clinical practice guidelines

**DOI:** 10.1038/s41598-021-92715-w

**Published:** 2021-06-24

**Authors:** Supachai Ekwattanakit, Chattree Hantaweepant, Archrob Khuhapinant, Noppadol Siritanaratkul, Vip Viprakasit

**Affiliations:** 1 Siriraj Thalassemia Center, Faculty of Medicine Siriraj Hospital, Mahidol University, Bangkok, Thailand; 2 Division of Hematology, Department of Medicine, Faculty of Medicine Siriraj Hospital, Mahidol University, Bangkok, Thailand; 3 Division of Hematology & Oncology, Department of Pediatrics & Siriraj Thalassemia Center, Faculty of Medicine Siriraj Hospital, Mahidol University, 2 Wanglang Road, Bangkoknoi, Bangkok, 10700 Thailand

**Keywords:** Anaemia, Epidemiology

## Abstract

Based on Thalassemia International Federation clinical practice guidelines (CPG) for non-transfusion dependent and transfusion dependent thalassemia, several measures should be routinely implemented such as monitoring and surveillance of thalassemia related complications for early detection and proper clinical management. To evaluate the prevalence and the performance of routine surveillance for thalassemia related complications during 2 periods; before and after published CPGs (2012–2014 vs 2015–2017), data from 524 adult thalassemia patients attended at Siriraj hospital were compared among different treating physician groups; thalassemia, private hematology, and internal medicine clinics. Three most common complications were osteopenia/osteoporosis (69.8%), gallstones (67.6%) and abnormal vitamin D level (67.6%). Iron overload has been widely evaluated (93.1%) followed by liver function test (82.3%). However, the rate of evaluation for other complications were significantly reduced and < 25% of patients were evaluated in several complications. Comparing among clinics, the surveillance rate has increased significantly for several endocrine complications only in patients treated at thalassemia clinic but not in others. This study was the first study that evaluated real-world practical management of thalassemia patient in terms of complication surveillance. This different clinical practice has called for an immediate policy change to improve and standardize a care for thalassemia patients in Thailand.

## Introduction

Thalassemia syndromes are the most common hereditary hemolytic anemia worldwide^1^. Since 2013, Thalassemia International Federation (TIF) has launched new standard clinical practice guidelines (CPG) for non-transfusion dependent thalassemia (NTDT)^2^ and transfusion dependent thalassemia (TDT)^3^. Based on these guidelines, several measures should be routinely implemented, such as monitoring of iron overload (IOL) and surveillance of thalassemia related complications to detect such complications for early clinical management.

General recommendation for evaluation thalassemia related complications by TIF-CPG for TDT patients includes the following items: (1) IOL by monitoring ferritin every 3 months and liver iron (by liver magnetic resonance imaging (MRI) after 8 years old or biopsy only if histology is required due to presence of hepatitis) every 12 months, (2) liver function and disease by liver function test every 3 months and yearly hepatitis profile (hepatitis A, B, and C serology) and ultrasonography, (3) endocrinopathy including thyroid function test, parathyroid hormone, glucose tolerance test (yearly) and calcium and fasting glucose every 3 months, (4) osteoporosis by vitamin D level every 3 months and bone mineral density (BMD) every 24 months, and (5) cardiac complications by yearly electrocardiogram, echocardiography and cardiac MRI^3^. For NTDT patients, similar assessments for IOL, liver function and disease, endocrinopathy, and bone disease are also recommended in TIF-CPG for NTDT^2^. While adrenocorticotropic hormone stimulation test was recommended for yearly monitoring of adrenal insufficiency in NTDT population^2^.

In Thailand alone, over 600,000 patients with different thalassemia types are expected^4^, and hematologists are currently treating not all of them, most of them are under the care of internists or general practitioners (GP). At present, there is no data in Thailand on how internists and hematologists are treating thalassemia patients in real-world practice. In particular, how early thalassemia related complications are currently evaluated to follow those of both guidelines. This study was aimed to evaluate routine care regarding surveillance for thalassemia related complications comparing two periods, before and after two TIF-CPG were published, and their prevalence throughout study period in patients at Siriraj hospital in Bangkok.

## Results

### Basic characteristics of study population

Total 459 NTDT (87.6%) and 65 TDT (12.4%) adult patients who were consecutively followed up during the study periods (2012–2014 and/or 2015–2017) were studied. The median number of transfusion episodes in NTDT patients during 2012–2014 and 2015–2017 were 0 episodes (interquartile range [IQR] = 0–1) and 0 episodes (IQR = 0–0), respectively. Current basic characteristics were shown in Table 1. None of TDT patients were treated at the internal medicine clinic (Table 1). All study patients had average age of 41.2 ± 16.2 years, mean Hb of 79 ± 15 g/L, and mean ferritin of 1034 ± 1327 μg/L There were statistically significant differences (p < 0.05) in age and Hb between NTDT patients in different treatment groups (Thal, Private, and Non-Heme), but no differences between demographic data among TDT patients in different treatment groups (Table 1). The majority of TDT patients in both Thal and Private clinics were β-thalassemia (98.2% and 100%, respectively), which are mainly Hb E/β-thalassemia disease (89.3% and 87.5%, respectively, Table 1). While there were more β-thalassemia NTDT patients (64.8%) in Thal clinic compared to Private and Non-heme clinics (37.6% and 34.7%, respectively, Table 1). Most α-thalassemia patients were deletional Hb H disease (68.7%) and non-deletional Hb H disease (30.9%).

**Table 1:** Recent basic characteristics of the study population.

Characteristics	NTDT			TDT			Total
	Thal	Private	Non-heme^c^	Thal	Private	Non-heme	
No. of patients (total)	459			65			
During the 2012–2014 period	144	192	60	53	8	0	468
During the 2015–2017 period	153	90	52	51	7	0	453
Age (years; mean (SD))	35.3 (12.3)	45.6^a^ (17.4)	52.5^b^ (13.9)	30.4 (6.4)	33.6 (5.7)	–	41.2 (16.2)
**Type of thalassemia (%)**							
α-thalassemia	35.2	62.4	65.3	1.8	0	0	47.5
Deletional Hb H disease^d^	*54.1*	*73.5*	*88.9*	*100*	–	–	*68.7*
Non-deletional Hb H disease^d^	*44.7*	*26.5*	*11.1*	–	–	–	*30.9*
β-thalassemia	64.8	37.6	34.7	98.2	100	0	52.5
Hb E/β-thalassemia^e^	*96.6*	*90.1*	*91.7*	*89.3*	*87.5*	–	*92.0*
Homozygous β-thalassemia^e^	*3.4*	*9.9*	*8.3*	*10.7*	*12.5*	–	*7.3*
Hemoglobin (g/L; mean (SD))	73 (13)	82^a^ (15)	91^b^ (14)	73 (13)	65 (19)	–	79 (15)
Ferritin (μg/L; mean (SD))	886 (799)	617^a^ (494)	761 (737)	2951 (2419)	1478 (968)	–	1034 (1327)

^a,b^*p* < 0.05 comparing between Thal vs. Private and Thal vs. Non-heme groups, respectively.

^c^No available ferritin in 33 patients.

^d,e^The percentage within α-thalassemia and β-thalassemia, respectively.

### Prevalence and overall surveillance rate of thalassemia related complications

**Figure 1:**
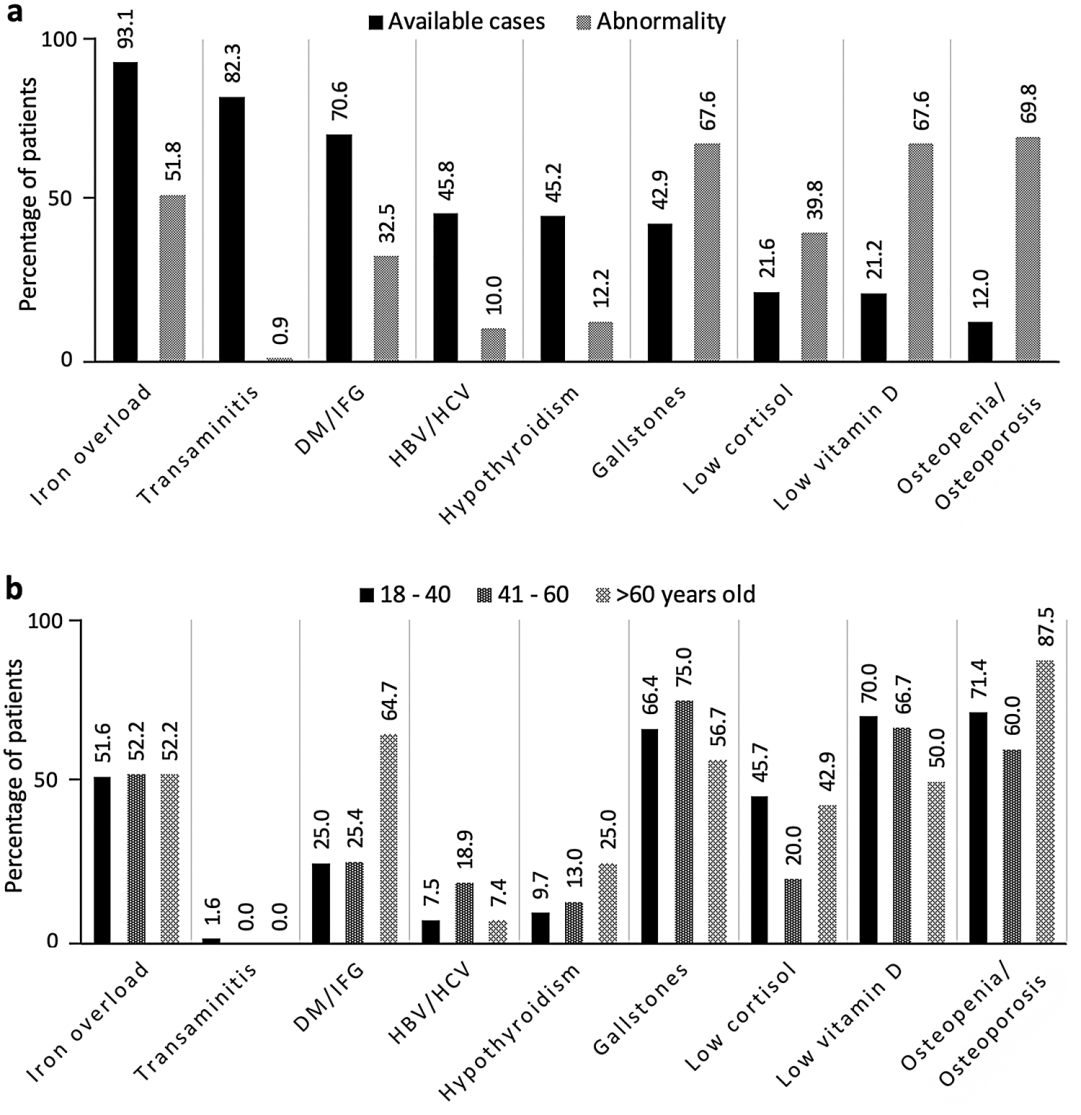
Complication of thalassemia patients throughout a study period (2012–2017). (**a**) Percentage of available patients that have been tested and those that have been diagnosed for each complication. (**b**) Rates of patients with each complication by age group.

In order to record prevalence of thalassemia related complications in study population, all available laboratory and radiological results from all patients throughout the study period (2012–2017) were evaluated. The three most common complications in available patients were osteopenia/osteoporosis (69.8%), gallstones (67.6%), and abnormal vitamin D levels (67.6%, Fig. 1a). IOL has been widely evaluated in 93.1% of thalassemia cases followed by evaluation of liver function (82.3%). In most patients, serum ferritin was used for evaluation of IOL, while MRI for liver and/or cardiac IOL assessment has been performed only in 11.3% and 55.4% of NTDT and TDT patients, respectively. The rate of assessment for endocrine complications such as DM/IFG, hypothyroid, low cortisol status, low vitamin D level, gallstones, and virological surveillance for hepatitis B and C were significantly reduced, and less than 25% of patients were evaluated in several complications (Fig. 1a). Of note, only 12% of thalassemia patients were assessed for osteopenia/osteoporosis by BMD.

When comparing complication rates between different age groups (early adulthood; 18–40 years, middle adulthood; 41–60 years; and later adulthood; > 60 years), several complications including iron overload, gallstones, low vitamin D level, and osteopenia/osteoporosis were commonly found since early adulthood in available cases (> 50%, Fig. 1b).

**Figure 2:**
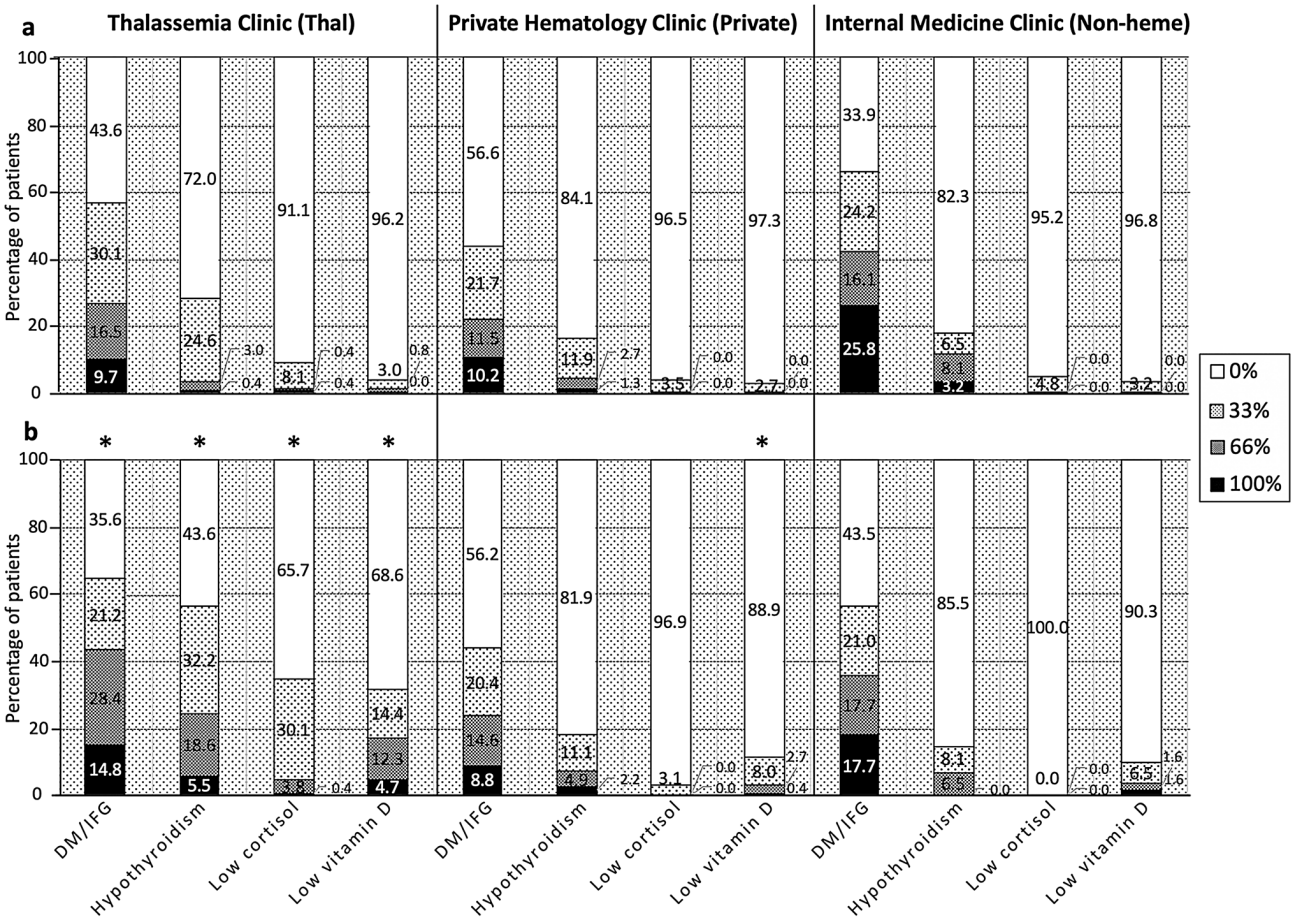
Comparing the frequency of yearly monitoring for thalassemia related complications; none (0%), one time (33%), 2 times (66%) and 3 times (100%) during three-year-period before CPG (2012–2014) (**a**) and after CPG (2015–2017) (**b**) by three different physician groups; thalassemia clinic, private hematology clinic, and non-hematology clinic. An asterisk indicates a significant difference between monitoring frequency before and after CPG (*p* < 0.05).

To test whether the implementation of TIF-CPG has any impact on our real-life clinical practice in terms of monitoring thalassemia related complications, the frequency of yearly monitoring for thalassemia related complications; none (0%), one time (33%), 2 times (66%) and 3 times (100%) during three-year-period before CPG (2012–2014) (Fig. 2a) and after CPG (2015–2017) (Fig. 2b) were evaluated among patients treated by 3 different treatment groups; Thal, Private, and Non-Heme clinics. In order to reduce bias, we decided to select endocrine complications including DM/IFG, hypothyroidism, low cortisol, and low vitamin D for this analysis as they are relatively inexpensive and do not required special assessment such as a specialist consultation. We found that the rate of routine complication surveillance has increased significantly for all evaluated endocrine complications in the group of patients treated at Thal clinic (*p* < 0.05), while only assessment of vitamin D level was significantly increased in Private clinic (Fig. 2). Interestingly, there was no change in Non-heme clinics.

To test whether the difference in complication monitoring rate was not affected by different proportion of thalassemia type as Thal clinic has more numbers of β-thalassemia patients compared to other clinics, we separately evaluated the result in both α-thalassemia and β-thalassemia groups in a similar analysis (Supplementary Figs. S1 and S2). The monitoring rates were significantly improved in both α-thalassemia and β-thalassemia patients in Thal clinic, except for the DM/IFG evaluation in α-thalassemia group.

## Discussion

This study was the first study that evaluated the real-world practical management of thalassemia patients in terms of complication surveillance. In adult thalassemia population, thalassemia related complications were not uncommon, and some occurred early in adulthood, which were consistent with the previous findings in Thai patients^5–9^. This result suggests that the prevalence of thalassemia related complications based on only available data from hospital records might be underestimating the true incidence of these complications and early detection of these complications is still lacking. For most complications, inexpensive laboratory/radiographic studies can screen them, and early detection could lead to appropriate treatment that can prevent other severe conditions and eventually improve patients’ quality of life. However, surveillance rates of these complications were low in all clinics, with the highest rate in the Thal clinic. After implement of thalassemia guidelines, the surveillance rates for all evaluated complications were increased only in patients treated at Thal clinic but not at other two clinics, suggesting that these two CPG have yet to be implemented by those physicians, and further endorsement is highly required.

Our data also emphasized a vital role of a specialized center toward better care for thalassemia patients. Such a specialized clinic required expertise and knowledge of responsible medical staff similar to our hematologists and thalassemia specialists described in our study. Previously, it has been shown that the survival outcome of Italian patients with thalassemia major has been dramatically improved compared to those treated in specialized centers and other centers^10^. Complying with clinical practice guideline is one of the critical measures any specialized center for thalassemia must follow and adopt into their routine practice.

There were limitations in our study. Firstly, assessment of iron overload was mainly performed by serum ferritin as only 16.8% of patients in this study were evaluated by MRI (data not shown). This was due to unavailability of MRI facility and high expense as this test became fully reimbursable for all patients after 2017. Secondly, this study could not record all thalassemia related complications as recommended in CPG, in particular cardiac complications, due to limited resource and availability of echocardiography as cardiologists were overwhelmed by referral patients with heart disease at our tertiary hospital. In most circumstance, thalassemia patients were sent to cardiology unit only when there is clinical clue for cardiac disease or significant cardiac iron overload. Lastly, a number of TDT patients were small as most patients have not been followed up longer enough (3 consecutive year period) at our center.

In summary, a two different standard of clinical practice even within the same tertiary care, such as Siriraj hospital, has called for an immediate policy change to improve and standardize care for thalassemia patients in Thailand. A standardized program for thalassemia patients based on these CPG for TDT and NTDT patients would provide an equal opportunity for all thalassemia patients to receive the same standard of care in the near future.

## Methods

### Study population

In this retrospective study, we analyzed data from a total of 3,233 adult thalassemia patients who were diagnosed and treated at our out-patient clinics at the Department of Medicine, Siriraj hospital, during 1994–2017. We divided them into two groups; 1 and 2 were those who have been treated and followed-up for three consecutive years during 2012–2014 and 2015–2017 (3 years before and after the implementation of thalassemia guidelines, respectively). This study was approved by Siriraj Institutional Review Board, Faculty of Medicine Siriraj Hospital, Mahidol University (No.499/2560(EC1)) and all research was performed in accordance with relevant guidelines/regulations. Informed consent from participants were waived by Siriraj Institutional Review Board due to retrospective data collection.

### Data collection

Clinical data and laboratory results, including age, types of thalassemia (NTDT, TDT, α- or β-thalassemia), recent steady-state hemoglobin (Hb) level, and transfusion status, were collected. NTDT was defined as those thalassemia patients who do not require regular transfusion for survival, except those who later on received regular transfusion due to thalassemia related complications, such as massive splenomegaly and pulmonary hypertension^11^. Complications and laboratory data (in parenthesis) were recorded including IOL (ferritin and, if available, liver iron concentration and cardiac T2* by MRI evaluation), significant transaminitis (ALT > 3 × upper limit of normal), diabetes mellitus (DM) or impaired fasting glucose (IFG) (fasting blood sugar), hypothyroidism (thyroid function test), low cortisol status (morning cortisol), vitamin D insufficiency/deficiency (total vitamin D), hepatitis B and hepatitis C infection (HBs Ag, anti-HCV and, if available, HCV RNA), osteopenia/osteoporosis (BMD) and gallstones (ultrasonography or computed tomography scan). To compare the care performance from different physician groups, patients were categorized into three treatment groups: (1) those who attended thalassemia clinic (Thal) treated mainly by hematologists, residents and clinical fellows in hematology, (2) those in private hematology clinic (Private) treated by mostly attending hematologists, and (3) those in the internal medicine clinic (Non-heme) treated by internists and GP.

### Statistics

Descriptive statistics (mean with percentage and standard deviation) were used for basic characteristics data. Comparisons of basic characteristics between treatment groups and yearly monitoring frequency of thalassemia related complications between three-year-period before CPG (2012–2014) and after CPG (2015–2017) were performed by Pearson Chi-square test. All *p-*values were 2-sided with the level of significance set at less than 0.05. Statistical analysis was performed using the SPSS software (version 20 for Windows; SPSS Inc, Chicago, IL, USA).

### Prior presentation

A part of this work was presented as a poster in the 59th Annual Meeting of American Society of Hematology held from December 9 to 12, 2017, in Georgia, Atlanta, USA.

### Ethics approval

This study was approved by the local ethical committee, Siriraj Institutional Review Board, Faculty of Medicine Siriraj Hospital, Mahidol University (No.499/2560(EC1)).

## Supplementary Information

Supplementary Information.
