# Supplementary material for: An urgent need for improving thalassemia care due to the wide gap in current real-life practice and clinical practice guidelines
Source: Sci Rep. 2021 Jun 24;11:13283. doi: 10.1038/s41598-021-92715-w (PMC8225847; doi:10.1038/s41598-021-92715-w)

**Supplementary Information**

# Title: An urgent need for improving thalassemia care due to the wide gap in current real-life practice and clinical practice guidelines

**Journal Name:** Scientific Reports

**Authors:** Supachai Ekwattanakit[a], Chattree Hantaweepant[b], Archrob Khuhapinant[b], Noppadol Siritanaratkul[b], Vip Viprakasit[a, c]

**Affiliations:**

[a]*Siriraj Thalassemia Center, Faculty of Medicine Siriraj Hospital, Mahidol University, Bangkok, Thailand*

[b]*Division of Hematology, Department of Medicine, Faculty of Medicine Siriraj Hospital, Mahidol University, Bangkok, Thailand*

[c]*Division of Hematology/Oncology, Department of Pediatrics, Faculty of Medicine Siriraj Hospital, Mahidol University, Bangkok, Thailand*

***Corresponding author:**

Vip Viprakasit, M.D., D.Phil. (Oxon)

Professor of Pediatrics

Director, Thalassemia Research Program

Division of Hematology & Oncology

Department of Pediatrics & Siriraj Thalassemia Center

Faculty of Medicine Siriraj Hospital

Mahidol University

2 Wanglang Road, Bangkoknoi

Bangkok 10700

THAILAND

Email: vip.vip@mahidol.edu

Telephone & fax: +66-24122113

Mobile: +66-858076859

**Figure captions**

**Supplementary Fig. 1** Comparing the frequency of yearly monitoring for thalassemia related complications in $\alpha$-thalassemia ($\alpha$-thal) patients; none (0%), one time (33%), 2 times (66%) and 3 times (100%) during three-year-period before CPG (2012-2014) (a) and after CPG (2015-2017) (b) by three different physician groups; thalassemia clinic, private hematology clinic, and non-hematology clinic. An asterisk indicates a significant difference between monitoring frequency before and after CPG ($p$ <0.05).

**Supplementary Fig. 2** Comparing the frequency of yearly monitoring for thalassemia related complications in β-thalassemia (β-thal) patients; none (0%), one time (33%), 2 times (66%) and 3 times (100%) during three-year-period before CPG (2012-2014) (a) and after CPG (2015-2017) (b) by three different physician groups; thalassemia clinic, private hematology clinic, and non-hematology clinic. An asterisk indicates a significant difference between monitoring frequency before and after CPG ($p$ <0.05).

## Supplementary Fig. 1

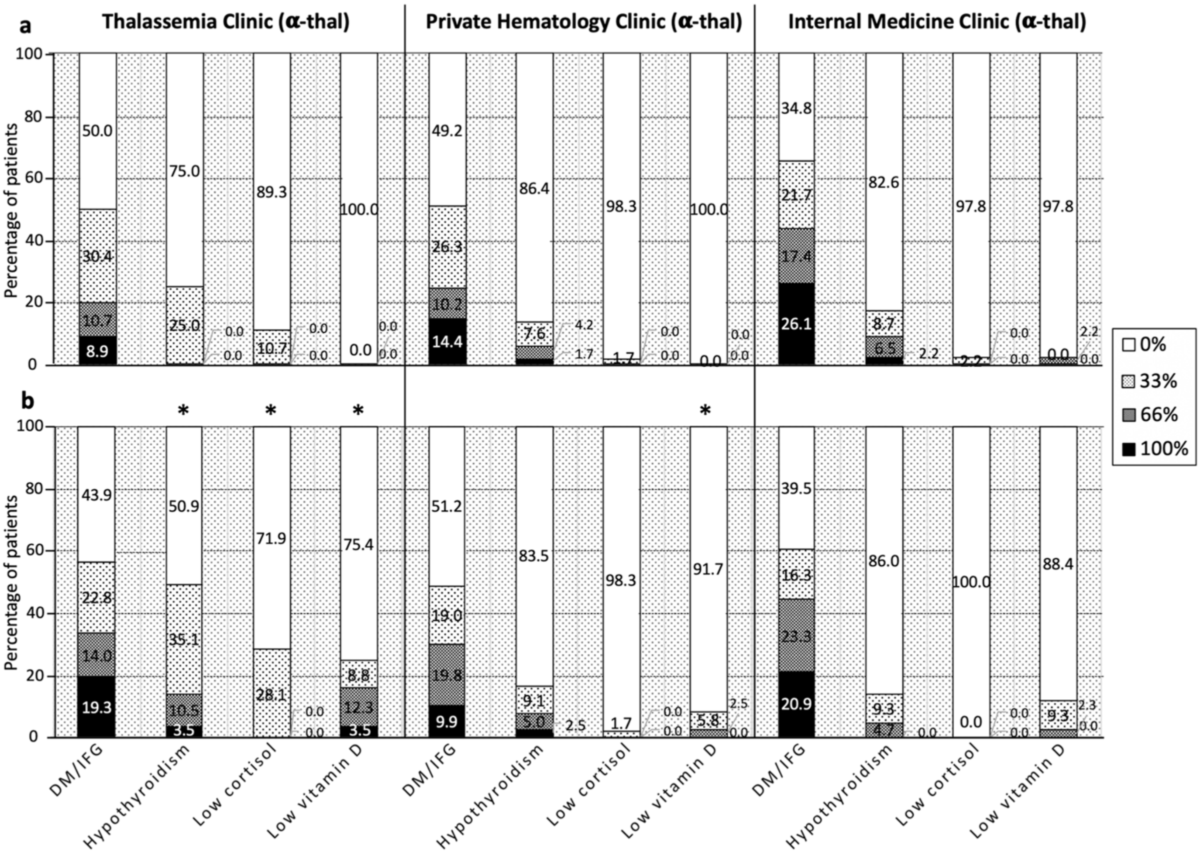

## Supplementary Fig. 2

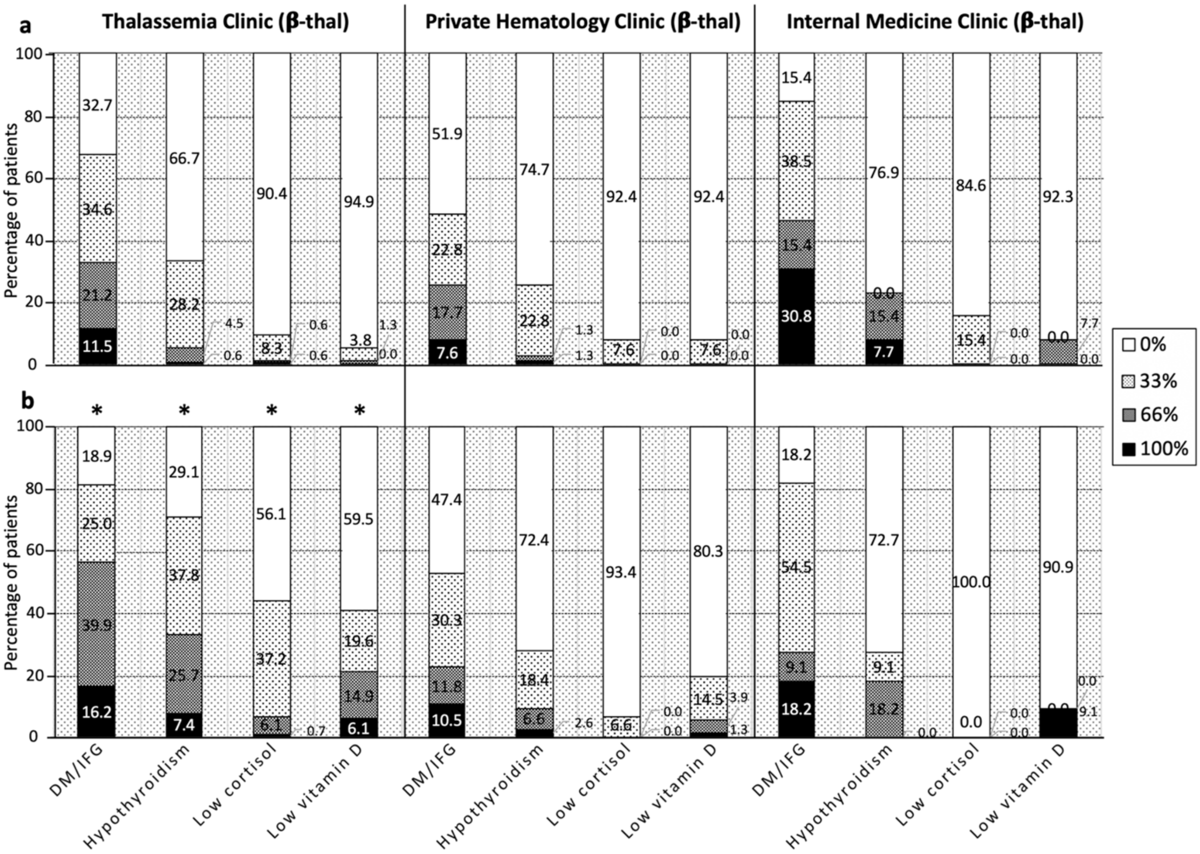

Supplement: Supplementary file 1 — Supplementary Information. [file 41598_2021_92715_MOESM1_ESM.pdf]
